# Niche-driven microbial architecture in mothers and newborns with minimal cohort influence across anatomically distinct sites

**DOI:** 10.3389/fimmu.2026.1896420

**Published:** 2026-07-17

**Authors:** Norbert Dera, Natalia Żeber-Lubecka, Joanna Ziemska-Legiecka, Magdalena Piątkowska, Aneta Balabas, Michał Ciebiera, Diana Massalska, Katarzyna Kosińska-Kaczyńska, Jerzy Ostrowski, Katarzyna Bubień, Tomasz Kozera, Kacper Dera, Iwona Szymusik

**Affiliations:** 1 Department of Obstetrics, Perinatology and Neonatology, Center of Postgraduate Medical Education, Warsaw, Poland; 2 Warsaw Institute of Women’s Health, Warsaw, Poland; 3 Department of Gastroenterology, Hepatology and Clinical Oncology, Centre of Postgraduate Medical Education, Warsaw, Poland; 4 Department of Experimental Oncology, Laboratory of Cancer Metabolism, Maria Sklodowska-Curie National Research Institute of Oncology, Warsaw, Poland; 5 Department of Gynecology, Obstetrics and Gynecological Endocrinology with a Genetic Laboratory, Centre of Postgraduate Medical Education, Warsaw, Poland; 6 Department of Pediatric Cardiology and General Pediatrics, Medical University of Warsaw, Warsaw, Poland

**Keywords:** fetus, microbiota, newborn, pregnancy, transmission

## Abstract

The microbiota plays a crucial role in the functioning of the human body. Several theories have been proposed regarding the origin of the uterine microbiota, including both ascending pathways from the vagina and translocation from the gastrointestinal tract. Numerous studies suggest that, in preterm births, amniotic fluid may contain microbial DNA or signals associated with microorganisms ascending from the vagina, even in the presence of intact fetal membranes, as well as by microbes originating from the placental microbiome.

**Background:**

The aim of this study was to comprehensively characterize the maternal and neonatal microbiome across multiple anatomically and biologically distinct niches and to determine the extent to which the structure of microbial communities reflects (i) anatomical location, (ii) maternal–infant relationships, and (iii) cohort-related perinatal factors.

**Methods:**

The study included eight women who delivered between 34 + 0 and 36 + 6 weeks of gestation, along with their neonates. The control group consisted of eight women who delivered at ≥37 + 0 weeks of gestation and their neonates. Prior to delivery, a cervical swab, an oral (buccal) swab, and a stool sample were collected. After delivery, the following samples were obtained from the neonate: a skin swab, a sample of amniotic fluid aspirated from the stomach, a rectal swab, and placental tissue. To assess microbiota composition, microbiome profiling based on 16S rRNA gene sequencing was performed.

**Results:**

The analysis did not demonstrate significant differences in microbiota composition between late preterm and term pregnancies, in either mothers or neonates.

**Conclusion:**

Comparative analysis of maternal and neonatal microbiota suggests a possible association with microbial signals consistent with an ascending vaginal contribution; however, without a detectable influence on neonatal microbiota. Conversely, the findings may indicate a potential association with maternal microbiota consistent with a hematogenous contribution, however, no direct evidence of microbial colonization or transmission can be inferred.

## 1 Introduction

The microbiota plays a crucial role in the functioning of the human body. It is involved in proper digestion, metabolic processes, trophic effects, and it also influences the development and maturation of the host’s innate and adaptive immune systems (1–4).

Several theories have been proposed regarding the origin of the uterine microbiota, including both ascending pathways from the vagina and translocation from the gut. The latter may occur via translocation of microbial components from intestinal vessels into the peritoneal cavity with subsequent reabsorption through the fallopian tubes, as well as through dendritic cells and leukocytes transporting microbial material hematogenously to the uterus (5).

Numerous studies suggest that amniotic fluid in preterm births may become colonized by microorganisms ascending from the vagina, even in the presence of intact fetal membranes, as well as by microbes originating from the placental microbiome (6). At the same time, the vagina may serve as a source of microorganisms that reach the placenta, amniotic fluid, and fetus via translocation across the chorionic villi (7, 8).

Earlier research methodologies, which did not distinguish between viable and non-viable bacteria and did not allow for simultaneous maternal blood sampling to determine whether the detected microbiome originated from maternal or fetal chorionic tissue, contributed to the prevailing view prior to 2014 that the placenta does not harbor its own microbiome (9). Current evidence suggests that bacterial infections may play a significant role in the pathogenesis of preterm premature rupture of membranes (PPROM) and preterm birth (10–13).

The aim of this study was to comprehensively characterize the maternal and neonatal microbiome across multiple anatomically and biologically distinct niches, and to determine the extent to which microbial community structure reflects (i) anatomical site, (ii) maternal-infant relationships, and (iii) cohort-related perinatal factors. By integrating alpha-diversity, beta-diversity and genus-level differential-abundance analyses, we sought to evaluate whether neonatal microbial communities resemble those of their anatomically corresponding maternal sites, and whether cohort stratification (late preterm vs term pregnancy) contributes meaningfully to microbial variation within and across low- and high-biomass niches. This focus on late-preterm and term pregnancies is particularly relevant because major microbiota-related differences reported in the literature typically concern infants born before 33 weeks of gestation, whereas systematic characterization of late-preterm microbiota across maternal and neonatal niches remains limited.

## 2 Materials and methods

### 2.1 Design

This prospective, observational, cross-sectional pilot cohort study was conducted between August 2023 and June 2024 at the Department of Neonatology and Neonatal Intensive Care of Bielanski Hospital, the Department of Obstetrics, Perinatology and Neonatology of the Centre of Postgraduate Medical Education (CMKP), the Department of Neonatology and Neonatal Pathology of the Warsaw Institute of Women’s Health, the Second Department of Obstetrics and Gynecology of CMKP, in collaboration with the Department of Gastroenterology, Hepatology and Clinical Oncology of CMKP. The study protocol involving human participants was approved by the Bioethics Committee of the Centre of Postgraduate Medical Education and was conducted in accordance with the approved protocol (No. 347/2023). Written informed consent was obtained from all mothers of the neonates participating in the study.

### 2.2 Patient population

The study group consisted of eight women with singleton pregnancies delivered between 34 + 0 and 36 + 6 weeks of gestation (late preterm, LP), along with their neonates. The control group (term pregnancy, TP) comprised eight women who delivered at ≥37 + 0 weeks of gestation, together with their neonates.

Inclusion criteria for the LP were as follows: (1) maternal age >18 years, (2) viable singleton pregnancy, (3) gestational age between 34 + 0 and 36 + 6 weeks (confirmed by first-trimester ultrasound examination), (4) absence of amniotic fluid leakage, (5) no use of antibiotics or probiotics within 4 weeks prior to study inclusion, and (6) elective delivery by cesarean section for either obstetric indications, including cord prolapse, eclampsia, imminent eclampsia, fetal heart rate abnormalities, malpresentation, and cephalopelvic disproportion (e.g., pelvic anatomical abnormalities or a thin uterine scar <2 mm), or non-obstetric indications, such as neurological, ophthalmological, pulmonary, orthopedic, hematological, cardiological, psychiatric, or other conditions.

Inclusion criteria for the TP cohort were consistent with those for the LP, except for gestational age, which was defined as 37 + 0 to 41 + 6 weeks (confirmed by first-trimester ultrasound examination). The inclusion of cesarean delivery was intended to minimize neonatal exposure to maternal vaginal microbiota during birth and thereby reduce delivery-mode–related variability.

Exclusion criteria were as follows: (1) inability to provide informed consent, (2) preterm premature rupture of membranes (PPROM), (3) premature rupture of membranes (PROM), (4) vaginal delivery, (5) multiple pregnancy, (6) intrauterine fetal demise, and (7) use of antibiotics or probiotics within 4 weeks prior to study inclusion.

### 2.3 Sample collection and DNA extraction

Biological material from both mothers and their neonates was collected at collaborating centers by physicians—experienced clinicians—in two stages.

Prior to delivery, a cervical swab (a routinely performed examination), a buccal swab, and a stool sample were obtained. Subsequently, after birth, the following samples were collected from the neonate: a skin swab, a sample of amniotic fluid evacuated from the stomach (using a suction catheter routinely employed for airway clearance), and a rectal swab (collected with a swab applicator during assessment of anal patency), as well as material derived from the placenta (collected after its delivery). To minimize the risk of contamination, immediately following the delivery during elective cesarean section, the placenta was transferred by the surgical team to a sterile instrument table positioned adjacent to the operating field. The investigator responsible for tissue collection was dressed in sterile surgical attire and wore sterile gloves. Placental tissue samples were then obtained using a sterile surgical scalpel (separate scalpel for maternal and fetal side). Specimens were first collected from the maternal (basal) surface of the placenta (from different cotyledons), followed by samples from the fetal (chorionic) surface. Basal and chorionic samples were stored separately. All collected tissue specimens were immediately secured and processed under sterile conditions to prevent contamination. Swab samples were collected using 4N6FLOQSwabs. The collected material was placed into Eppendorf-type tubes. Samples were obtained from sixteen mothers and their neonates (112 samples/assays), then immediately frozen and stored in ultra-low temperature freezers at approximately −70 °C.

Genomic DNA was isolated from all maternal and neonatal sampling sites using protocols optimized for the respective specimen types. Material obtained from maternal cervix, cheek, skin, and placental tissue, as well as neonatal stomach aspirates, rectal swabs, skin swabs, and placental fragments, was processed with the QIAamp DNA Mini Kit (Qiagen, Hilden, Germany) following the manufacturer’s instructions (14). DNA from maternal stool samples was extracted using the QIAamp Fast DNA Stool Mini Kit (Qiagen) (15). After extraction, DNA yield and purity were quantified fluorometrically with the Qubit dsDNA High Sensitivity Assay (Thermo Fisher Scientific, Carlsbad, CA, USA). All samples were stored at −20 °C until library preparation.

### 2.4 16S rRNA gene library preparation and sequencing

Bacterial community profiling was performed using a targeted 16S rRNA gene approach. Libraries were generated with the Ion 16S™ Metagenomics Kit, which amplifies multiple hypervariable regions of the 16S rRNA gene, in combination with the Ion Plus Fragment Library Kit (Thermo Fisher Scientific). Library preparation, adapter ligation, and barcoding were performed according to the protocol supplied by the manufacturer.

Sequencing was carried out on the Ion GeneStudio™ S5 System using Ion 510™, 520™, and 530™ chips, with run configuration and loading conditions optimized for low-biomass samples. To monitor potential contamination associated with low-biomass specimens (e.g., placenta, neonatal skin, neonatal stomach), extraction blanks and library blanks were included throughout the workflow and processed identically to biological samples. These controls were used to monitor background contamination and were incorporated into downstream decontamination analyses.

### 2.5 Bioinformatic processing and statistical analyses

Subsequent preprocessing was carried out in R (version 4.5.2) using the DADA2 package (version 1.38.0) (16). Raw reads were first filtered and trimmed with the filterAndTrim function, after which chimeric sequences were identified and removed using removeBimeraDenovo. Taxonomic assignment of the 16S rRNA gene sequences was performed against the SILVA reference database (version r138_2019) (17). To account for potential background contamination, decontamination was conducted with the micRoclean package (version 0.0.0.90), incorporating laboratory blank controls throughout the process [micRoclean: Microbial contamination removal toolkit. R package version 0.0.0.90.]. The resulting amplicon sequence variant (ASV) table was then imported into a phyloseq object to facilitate downstream analyses (18). All downstream analyses were primarily conducted at the genus taxonomic level, unless otherwise specified. Relative taxon abundances were derived after bioinformatic processing of sequencing reads and analyzed using compositional data approaches, including CLR transformation and differential abundance testing with ANCOM-BC2. Taxonomic composition analyses included all taxa identified after quality filtering and decontamination and were restricted by a prevalence threshold of 0.2 to reduce sparsity and improve robustness. For each sample type, we calculated a range of alpha diversity indices, including Shannon, Chao1, Simpson, Observed, and Fisher. Differences in alpha diversity between maternal and neonatal samples were evaluated using Wilcoxon tests. Beta diversity analyses were based on centered log-ratio (CLR)–transformed data generated with the microbiome package (version 1.32.0). We computed Aitchison distances and performed principal component analysis (PCA) to visualize compositional differences between groups. Group separation was tested using PERMANOVA (pairwiseAdonis package, version 0.4.1), and ANOSIM from vegan package was applied as an additional confirmatory method, yielding consistent outcomes [Martinez Arbizu P. pairwiseAdonis: Pairwise multilevel comparison using adonis. R package version 0.4.1.; Oksanen J et al., 2022. vegan: Community Ecology Package.]. Differential abundance was assessed using the ancombc2 function from the ANCOMBC package (version 2.12.0) (19). To reduce sparsity, we applied a prevalence cutoff (prv_cut) of 0.2, excluding taxa present in fewer than 20% of samples. The pseudo_sens option was disabled to improve model stability given the limited sample size. P-values were corrected for multiple testing using the Benjamini–Hochberg procedure (20).

Because of the small sample size, comparisons of alpha diversity between study groups were performed using the Mann–Whitney U test, while paired samples—when available—were analyzed with the Wilcoxon signed-rank test. Plots were generated with ggplot2 (version: 4.0.2), ggpubr (version: 0.6.3) and patchwork (version: 1.3.2) packages [ggplot2 (version 4.0.2) Wickham H. ggplot2: Elegant Graphics for Data Analysis. Springer-Verlag New York; 2016. https://ggplot2.tidyverse.org; ggpubr (version 0.6.3) Kassambara A. ggpubr: ‘ggplot2’ Based Publication Ready Plots. R package version 0.6.3. https://CRAN.R-project.org/package=ggpubr; patchwork (version 1.3.2) Pedersen TL. patchwork: The Composer of Plots. R package version 1.3.2. https://CRAN.R-project.org/package=patchwork].

## 3 Results

### 3.1 Cohort-stratified diversity and composition across maternal and neonatal niches

**Figure 1:**
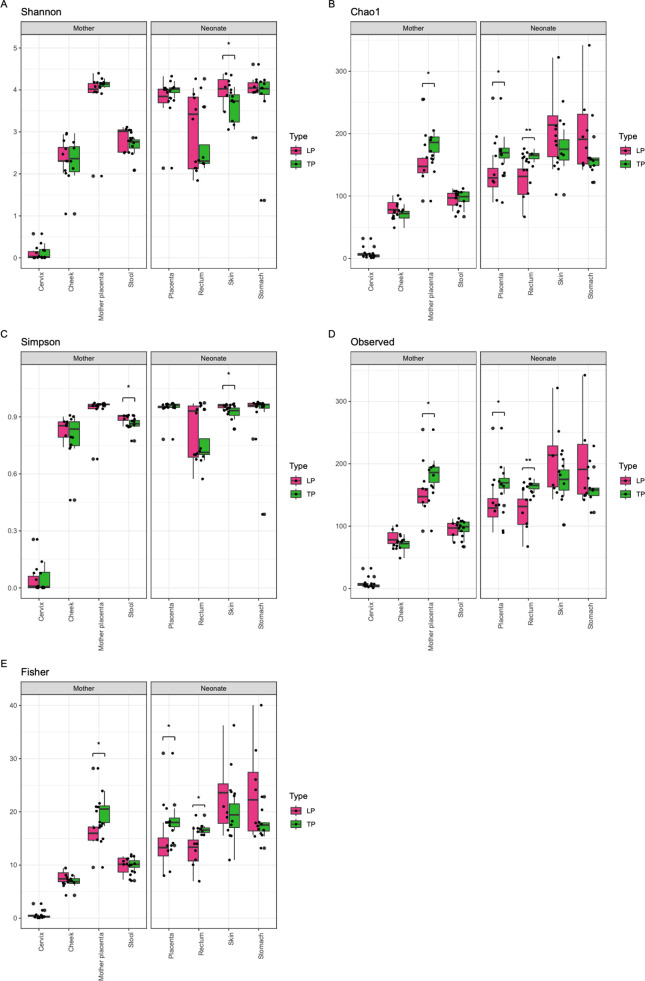
Alpha-diversity metrics across maternal and neonatal niches in late-preterm (LP) and term-pregnancy (TP) cohorts. **(A)** Shannon, **(B)** Chao1, **(C)** Simpson, **(D)** Observed ASVs, **(E)** Fisher’s alpha for maternal (cervix, cheek, maternal side of placenta, stool) and neonatal (rectum, skin, placenta, stomach) samples.

Alpha-diversity was quantified using richness-based (Observed ASVs, Chao1, Fisher’s alpha) and evenness-based (Shannon, Simpson) metrics. Across all maternal niches (cervix, cheek, maternal side of placenta, stool), no differences in alpha-diversity were detected between the LP and TP cohorts. All richness- and evenness-based indices showed nearly identical distributions between cohorts in every maternal site. Among newborn niches, rectum was the only site exhibiting a reproducible cohort effect, with significantly higher richness and evenness in cohort TP, supported by Chao1 (p = 0.0073), Simpson (p = 0.0073), and Fisher’s alpha (p = 0.00699) (**Figure 1**). Shannon and Observed measures were not significant. Skin showed a borderline difference in Observed richness (p = 0.0499), which did not replicate across other indices and would not remain significant after within-index correction (**Figure 1**). Placenta and stomach showed no LP-TP differences across any alpha-diversity metric (all p > 0.05) (**Figure 1**).

**Figure 2:**
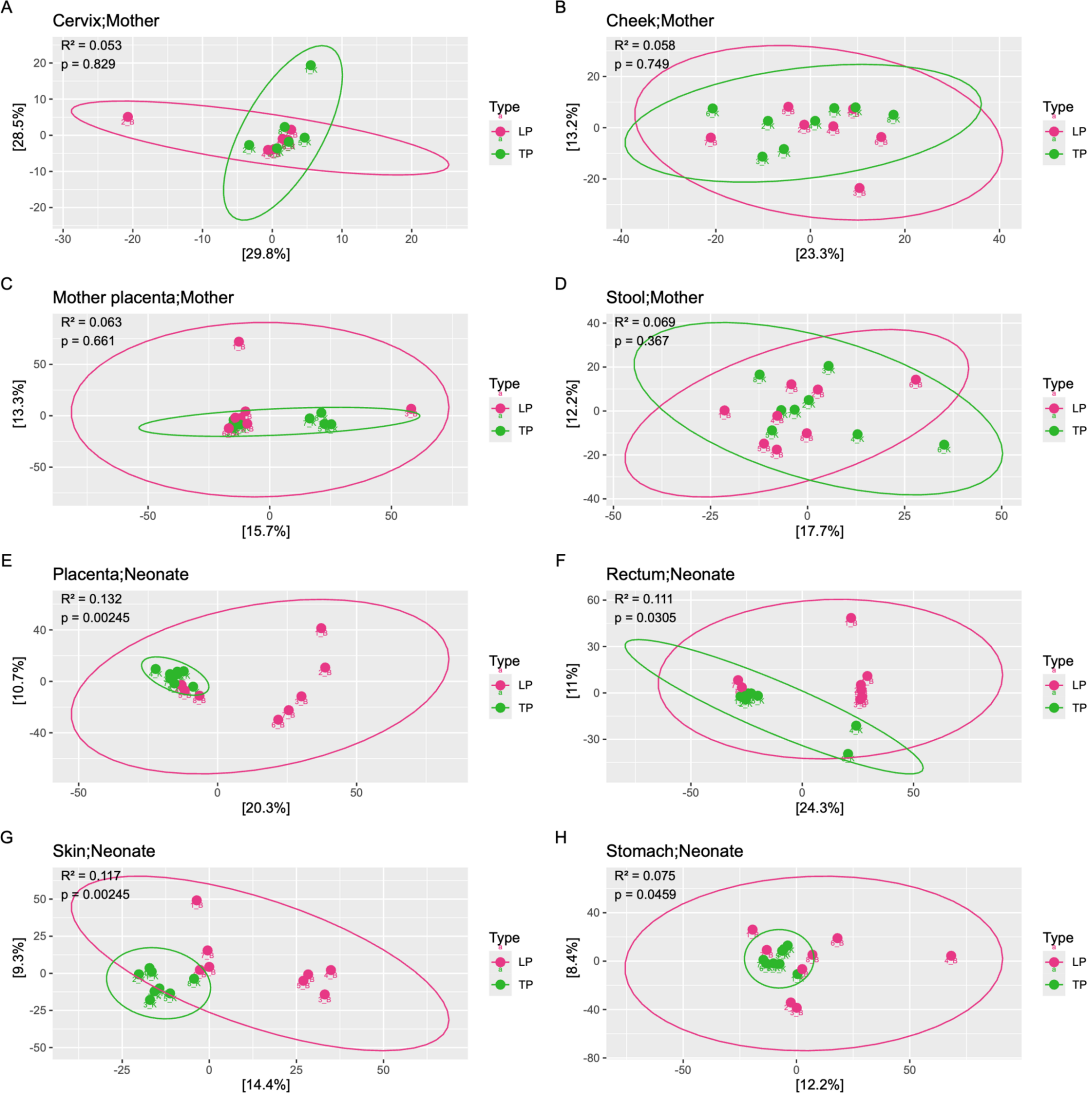
Beta-diversity of maternal and neonatal microbiota in late-preterm (LP) and term-pregnancy (TP) cohorts. PCoA based on Aitchison distance.

Beta-diversity analysis performed using PERMANOVA (adonis, Aitchison distance) demonstrated that most microbial niches differed significantly in community composition (p.adjust < 0.01 for nearly all pairwise comparisons). The largest dissimilarities were observed between physiologically distinct environments, including cheek vs. cervix (R^2^ = 0.646–0.676), cheek vs. stool (R^2^ = 0.563–0.595), and cervix vs. stool (R^2^ = 0.491–0.524), as well as placenta LP vs. cervix TP (R^2^ = 0.565) and placenta TP vs. stool TP (R^2^ = 0.525) (**Figure 2**). These high R^2^ values reflect differences between anatomical niches rather than between cohorts. Consistently, within each maternal niche no significant LP-TP differences were detected (cheek p.adjust = 0.728; cervix p.adjust = 0.84; stool p.adjust = 0.356; maternal placenta p.adjust = 0.658), confirming the absence of cohort-related effects (**Figure 2**). In contrast, newborn placenta of LP cohort and placenta TP differed significantly (p.adjust = 0.0025), whereas mother side of placenta of LP and TP cohort did not (p.adjust = 0.580) (**Figure 2**). PCoA visualizations corroborated the PERMANOVA results. Among maternal samples, niches exhibited clear separation and characteristic structural patterns: cervix showed the lowest dispersion with a compact cluster, cheek displayed the greatest heterogeneity, stool demonstrated high inter-individual variability without clustering, and mother placenta formed a coherent low-biomass cluster. In newborns, PCoA revealed two recurrent patterns of microbial organization, particularly evident in rectum, skin, and placenta, corresponding to low-biomass profiles and more diverse, colonized profiles. In stomach, a broad gradient was observed, reflecting substantial variability driven by perinatal exposure. All neonatal niches differed significantly from one another and from maternal niches (p.adjust < 0.01). Global relationships among all maternal and neonatal niches, independent of cohort, are further illustrated by PCA analysis (**Supplementary Figure 1**).

#### 3.1.1 Taxonomic composition

**Figure 3:**
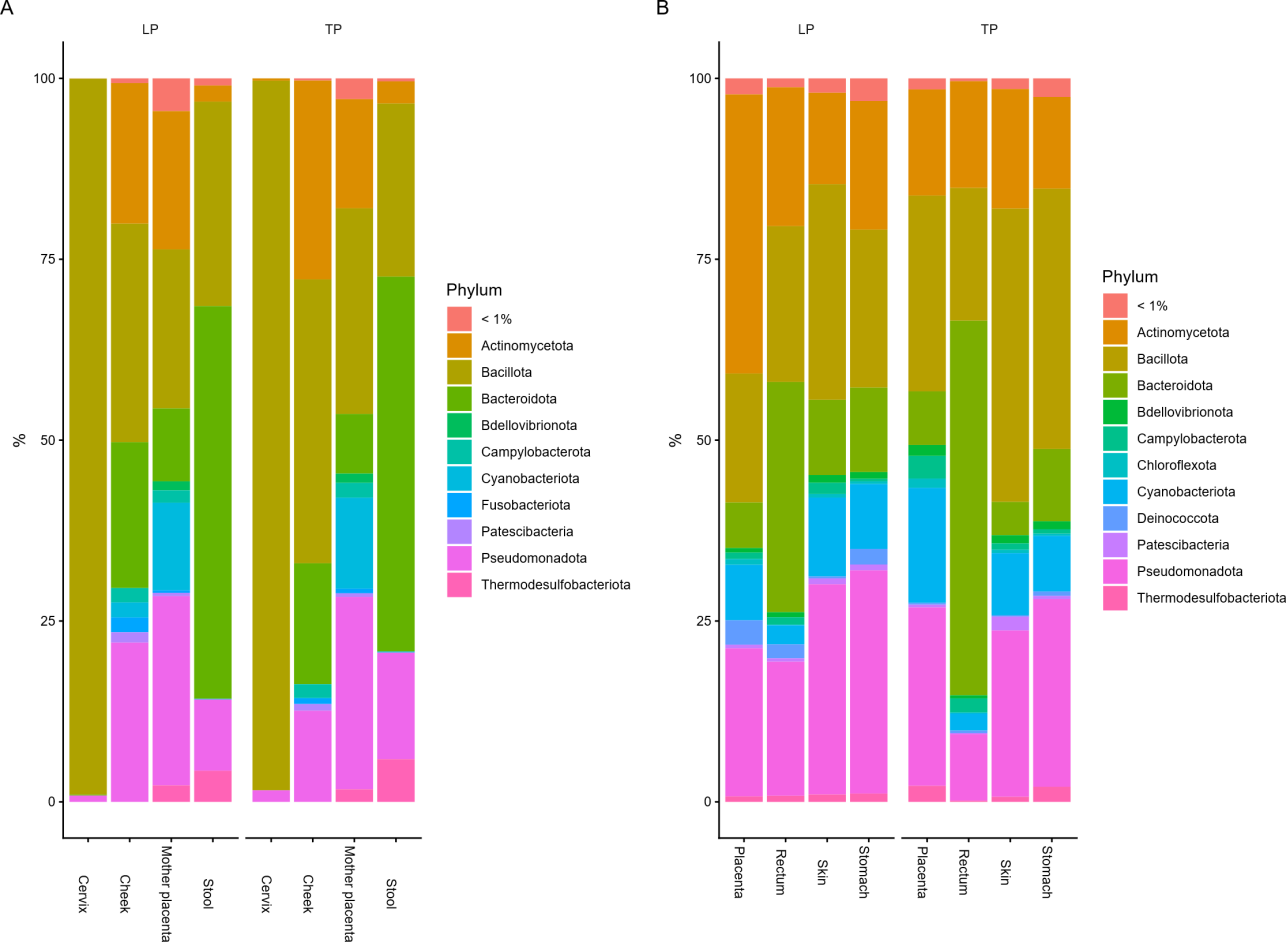
Phylum-level taxonomic composition in late-preterm (LP) and term-pregnancy (TP) cohorts. **(A)** Maternal samples; **(B)** Neonatal samples. ns: p ≥ 0.05: the difference is not statistically significant, *: p < 0.05: the difference is statistically significant., ***: p < 0.001: the difference is highly statistically significant.

To improve robustness and reduce noise associated with low-biomass samples, higher-level taxonomic summaries were also evaluated at the phylum level. Across maternal niches, the microbial community structure was dominated by a limited set of phyla, with proportions largely consistent between sequencing cohorts LP and TP. Bacillota (Firmicutes) and Actinobacteriota (Actinobacteria) represented the major components of the cervical and cheek microbiota, with minimal contribution from other phyla (**Figure 3A**). Maternal placental samples displayed a more heterogeneous profile, characterized by the presence of Pseudomonadota (Proteobacteria), Bacillota, and Bacteroidota (Bacteroidetes), accompanied by smaller fractions of low-abundance phyla. As expected, stool samples were enriched in Bacteroidota and Bacillota, with additional contributions from Actinobacteriota and minor phyla typical of adult gut microbiota. No systematic differences were observed between cohorts LP and TP in any maternal niche, indicating highly comparable taxonomic profiles across technical batches.

Newborn samples exhibited more diverse phylum-level patterns, varying substantially by anatomical site. Placental samples from newborns’ side showed mixed phylum composition, with contributions from Bacillota, Pseudomonadota, and Bacteroidota, along with low-abundance groups (**Figure 3B**). Rectal samples were dominated by Bacillota, Pseudomonadota, and Actinobacteriota, while skin samples showed prominent representation of Bacillota, Pseudomonadota, and Bacteroidota, consistent with early-life cutaneous microbial signatures. Stomach samples exhibited the highest heterogeneity among neonatal niches, with substantial proportions of Bacillota, Pseudomonadota, and Bacteroidota, along with a broader distribution of lower-abundance phyla. As in maternal samples, no meaningful differences in phylum-level community structure were detected between cohorts LP and TP within any neonatal site.

#### 3.1.2 Differential-abundance patterns across cohorts in low-biomass samples

Across the three low-biomass niches in which cohort contrasts were evaluated (maternal side of placenta, neonatal skin, neonatal stomach), genus-level differential abundance analysis (p adj < 0.1) yielded only sparse and heterogeneous signals, with no coherent or biologically interpretable pattern. In maternal placental samples, a small number of genera were statistically enriched in the TP cohort, but these consisted exclusively of environment-associated or low-biomass-prone lineages (e.g., *Gemmatimonas, Chryseolinea, Rhizobacter*), which are commonly detected at very low read counts in placental studies. Some of these taxa, including *Methylobacterium, Cupriavidus, Rheinheimera*, and *Deinococcus*, are also frequently reported in the literature as potential contaminants in low-biomass microbiome analyses. These observations do not imply active colonization but instead reflect low-abundance microbial signals typical of such environments, which may represent a mixture of true biological signal and background contamination. Neonatal skin showed a single cohort-associated genus (*Roseateles*), also an environmental lineage frequently observed in low-biomass contexts. Neonatal stomach exhibited a broad, taxonomically mixed set of low-abundance genera spanning environmental, gut-associated, and oral/skin-associated groups, without a unified or reproducible cohort-specific signature.

Importantly, no genera passed the p adj < 0.1 threshold in any of the remaining anatomical sites, including maternal cervix, cheek, and stool, as well as neonatal rectum and neonatal side of placenta, where feature-level statistics showed symmetric, low-effect distributions. Full differential-abundance results are therefore presented for completeness only (**Supplementary Table 1**), without inferring functional or ecological significance.

### 3.2 Mother-newborn niche architecture

To further explore mother-infant microbial relationships, we analyzed four biologically motivated cross-site contrasts representing the main potential axes of maternal-neonatal exposure: mother-stool vs. newborn-rectum (gut-gut axis); mother-cervix vs. newborn-placenta (birth-canal exposure axis); mother-cheek vs. newborn-stomach (oral-gastric axis), and mother-placenta vs. newborn-placenta (placental niche axis). These pairings reflect anatomically or exposure-related pathways through which microbial similarity could, in principle, arise.

**Figure 4:**
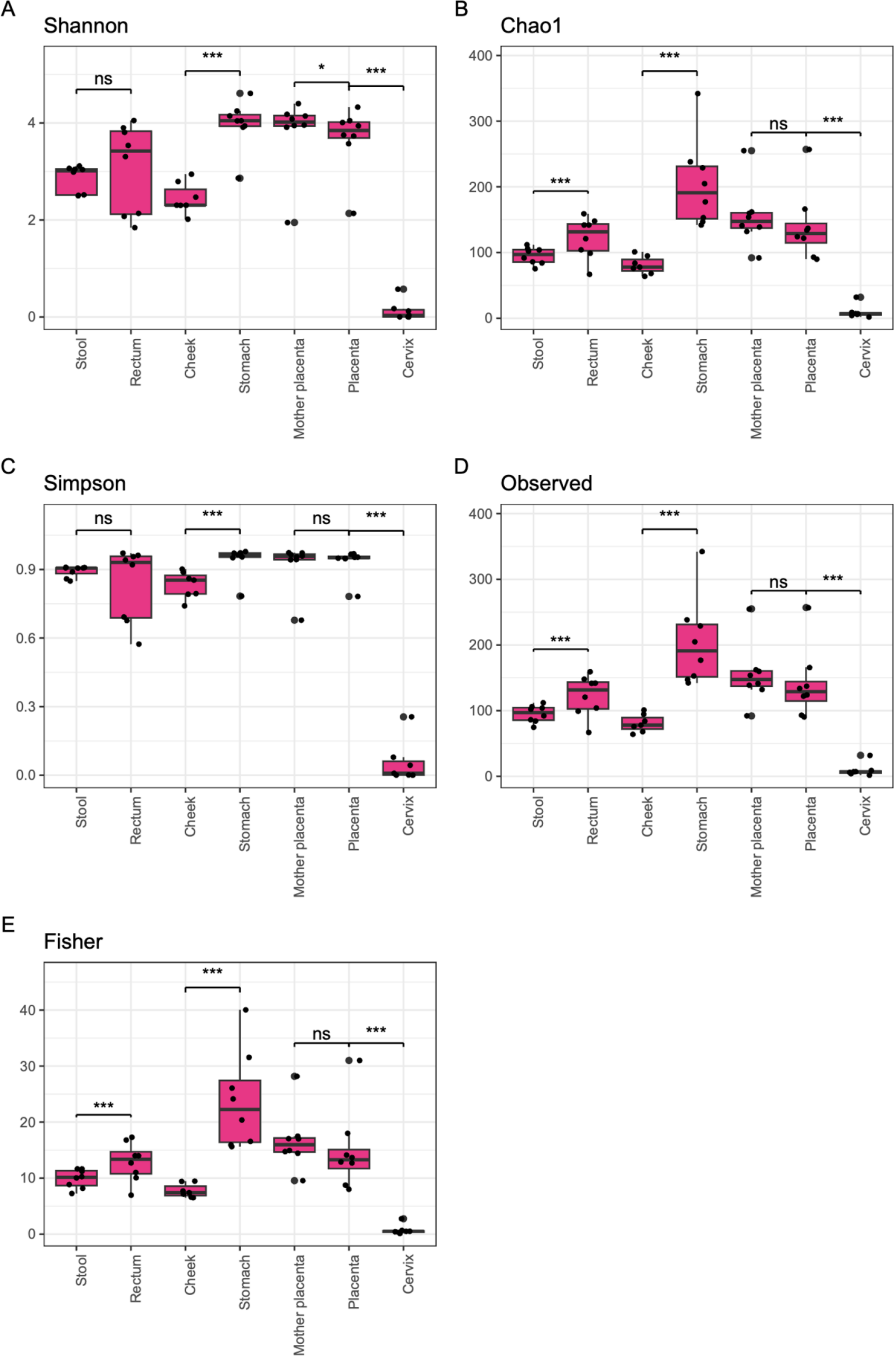
Alpha-diversity comparisons across mother-newborn niche pairs in late-preterm (LP) cohort.

**Figure 5:**
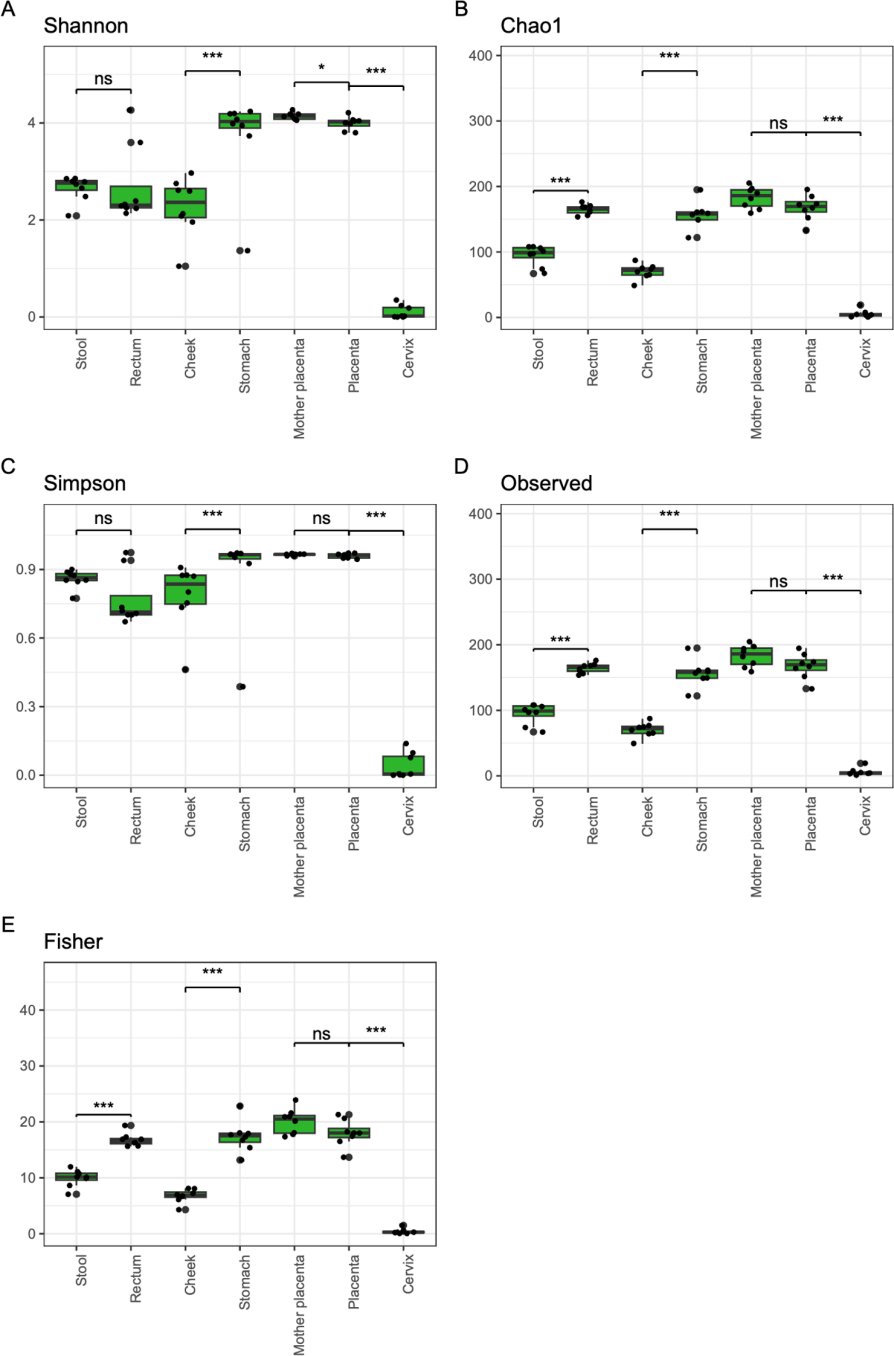
Alpha-diversity comparisons across mother-newborn niche pairs in term-pregnancy (TP) cohort. ns: p ≥ 0.05: the difference is not statistically significant, *: p < 0.05: the difference is statistically significant., ***: p < 0.001: the difference is highly statistically significant.

Alpha-diversity comparisons across mother-newborn niche pairs revealed clear, site-driven differences that were consistent across both sequencing cohorts. Maternal stool displayed the highest and most mature richness and evenness, whereas neonatal rectum showed substantially lower and more variable alpha-diversity. These differences were statistically significant in cohort TP for multiple indices (Chao1 p = 0.000915, Simpson p = 0.000915, Fisher p = 0.000155), while in cohort LP they manifested only as non-significant trends (Chao1 p = 0.0738, Simpson p = 0.0738, Fisher p = 0.0830) (**Figure 4**). A similarly consistent pattern emerged in the comparison between mother-cervix and newborn-placenta. The cervix, characterized by extremely low richness and evenness, differed markedly from neonatal placental samples, which exhibited higher but heterogeneous alpha-diversity; all indices were highly significant in both cohorts (Chao1 p = 0.00145 in LP; p = 0.000923 in TP; Shannon p = 0.00031 in LP and p = 0.000931 in TP) (**Figures 4**, **5**). In the mother-cheek vs. newborn-stomach comparison, maternal cheek showed a stable adult oral diversity profile, whereas neonatal stomach presented a broad range of richness and evenness values, reflecting early-life exposure. Here, all alpha-diversity indices were significantly higher in stomach than cheek in both cohorts (Chao1 p = 0.000311 in LP; p = 0.000923 in TP; Shannon p = 0.000622 in LP; p = 0.00699 in TP) (**Figures 4**, **5**). Finally, the placenta-mother vs. newborn-placenta; comparison reflecting two inherently low-biomass niches showed only minimal divergence in alpha-diversity. Differences were not significant in cohort LP across all metrics, and in cohort TP only Shannon reached nominal significance (p = 0.0104) without replication in other indices (**Figures 4**, **5**).

**Figure 6:**
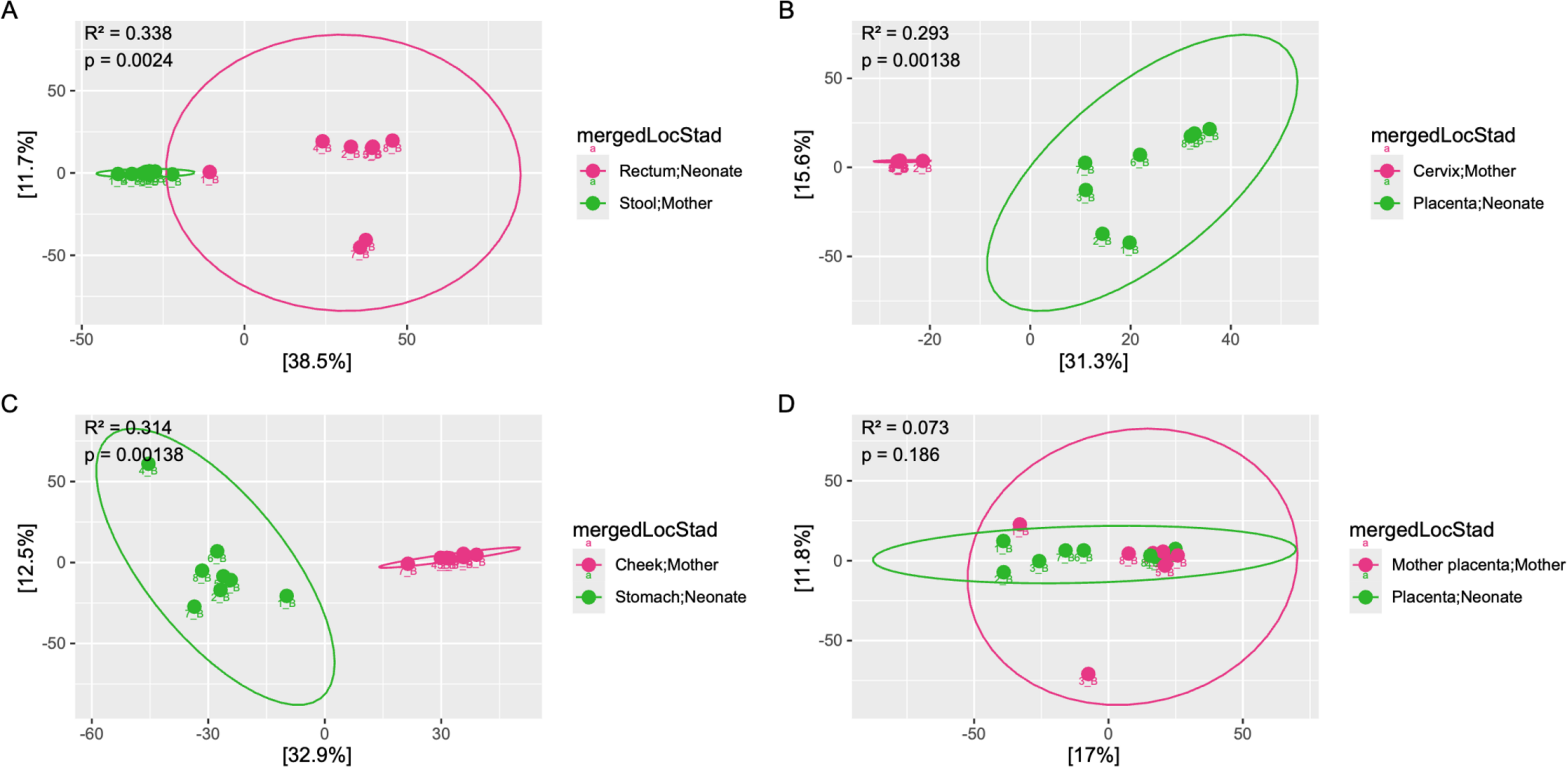
Beta-diversity of mother-newborn niche pairs in late-preterm (LP) cohort. PCoA (Aitchison distance) illustrating four mother-infant microbial axes.

**Figure 7:**
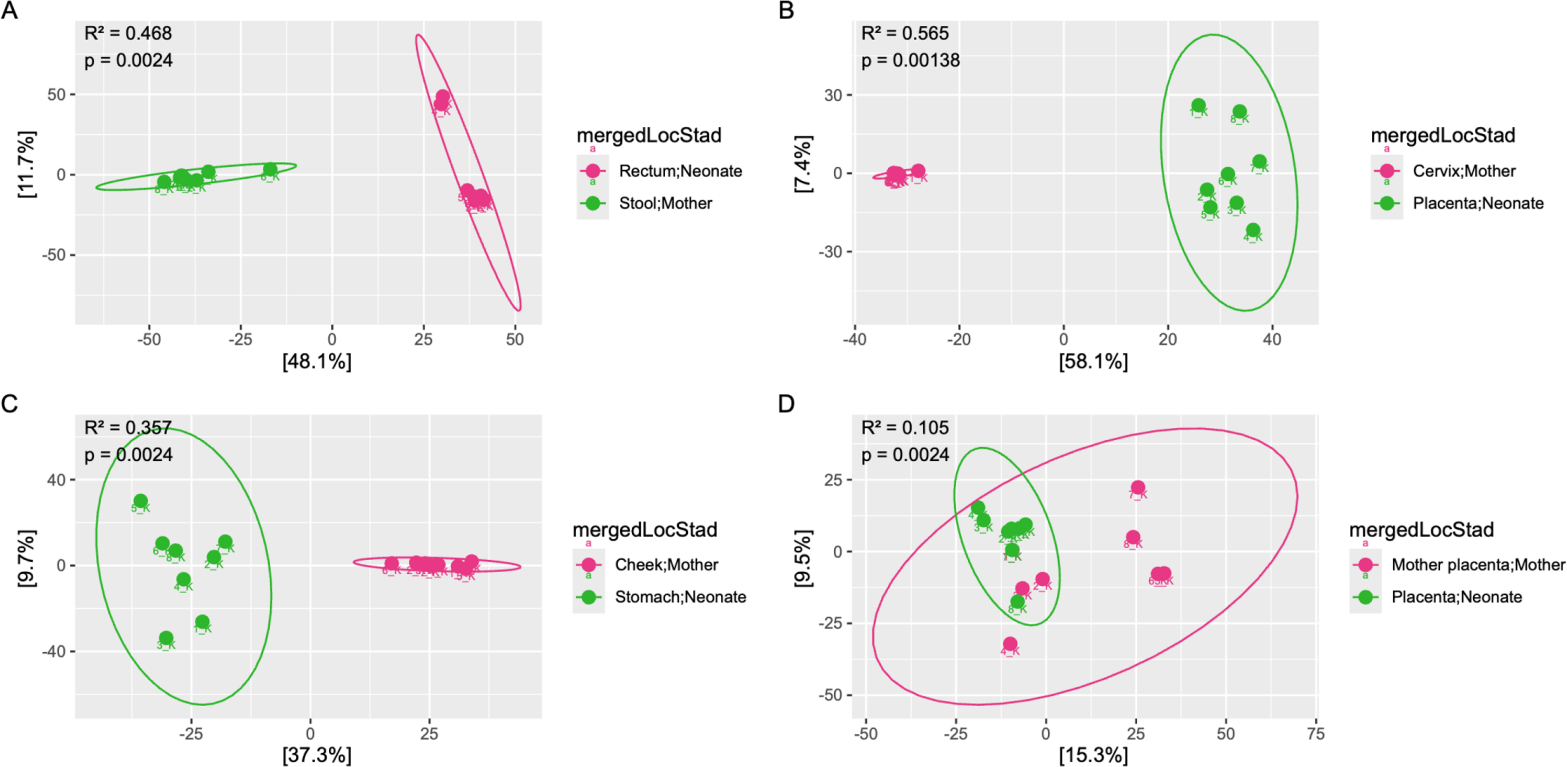
Beta-diversity of mother-newborn niche pairs in term-pregnancy (TP) cohort. PCoA (Aitchison distance) illustrating four mother-infant microbial axes.

PERMANOVA confirmed site-driven separation for all four mother-newborn contrasts (site R^2^ range ≈ 0.105–0.565), with every comparison significant (p.adjust < 0.01) except the low-biomass mother-placenta LP cohort (R^2^ ≈ 0.073; p.adjust = 0.199) (**Figures 6**, **7**).

#### 3.2.1 Differential-abundance profiles across mother-newborn niche pairs

Differential-abundance (DA) analysis across the three biologically motivated mother–newborn niche contrasts revealed distinct, niche-specific taxonomic shifts, with no evidence of direct maternal-infant similarity at the genus level. Each comparison exhibited a characteristic structure driven by the ecological properties of the respective tissues, and where applicable by the low-biomass nature of newborn samples.

**Figure 8:**
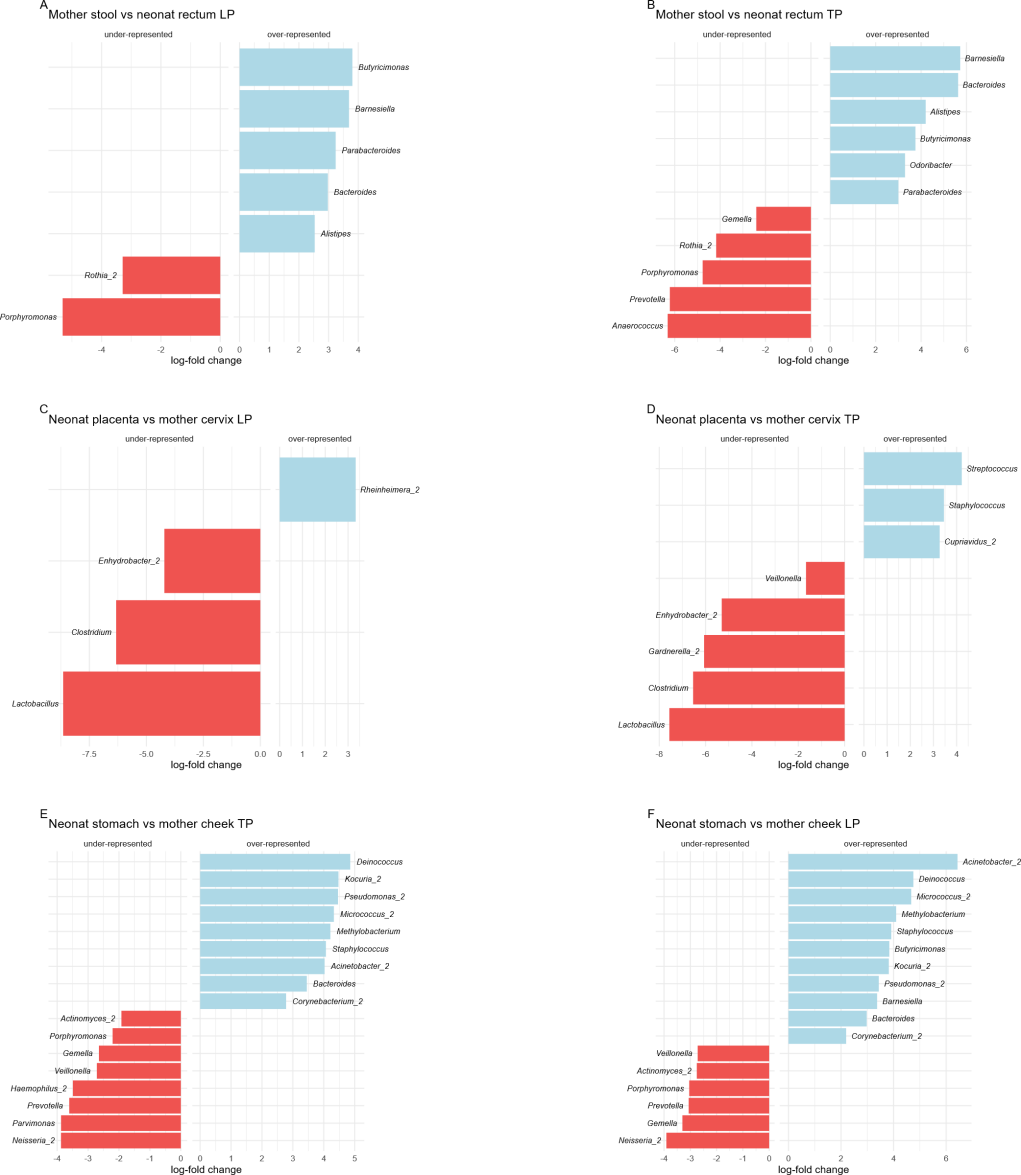
Genus-level differential-abundance profiles across mother-newborn niche pairs in late-preterm (LP) and term-pregnancy (TP) cohorts. Panels show DA results (log-fold change) for four biologically defined mother-infant contrasts. Blue bars indicate taxa enriched (positive log-fold change), whereas red bars indicate taxa depleted (negative log-fold change).

DA profiles clearly reflected the mature adult gut signature in maternal stool versus the immature, early-life profile of the neonatal rectum. In both cohorts, maternal stool was enriched in canonical gut genera, including *Bacteroides, Barnesiella, Alistipes, Parabacteroides, Butyricimonas*, and *Odoribacter* (p adj< 0.1). Conversely, genera enriched in the neonatal rectum comprised low-abundance, early-colonizing or opportunistic/environment-associated lineages, such as *Rothia, Porphyromonas, Anaerococcus, Prevotella, Gemella* and multiple Incertae Sedis groups, with no hallmark adult gut taxa overrepresented in newborns (**Figure 8**). These patterns were consistent across both LP and TP cohorts.

Differences between the neonatal placenta and maternal cervix were dominated by the *Lactobacillus*-rich profile of the adult cervix. *Lactobacillus* exhibited the strongest negative log-fold change in both cohorts (logFC ≈ −8 in LP; −7.6 in TP; p adj < 0.001), together with enrichments of *Clostridium, Enhydrobacter, Veillonella*, and *Gardnerella* in cervix. In contrast, genera enriched in the neonatal placenta consisted mainly of heterogeneous, low-biomass–prone or environment-associated lineages (e.g., Incertae Sedis groups, *Rheinheimera, Cupriavidus, Staphylococcus, Streptococcus*) appearing at low relative abundances and lacking any coherent ecological signature (**Figure 8**). These findings align with the low-biomass behavior of placental tissue and do not imply stable, niche-specific microbial signal.

DA patterns mirrored the exposure-driven gradient characteristic of the neonatal stomach. Maternal cheek displayed a consistent adult oral core *Prevotella, Neisseria, Porphyromonas, Veillonella, Haemophilus, Actinomyces, Gemella, Parvimonas* which was significantly enriched relative to stomach (p adj < 0.1). In contrast, the neonatal stomach was enriched in a mixed assemblage spanning environmental/opportunistic genera (*Acinetobacter, Pseudomonas, Methylobacterium, Deinococcus*), skin-associated taxa (*Staphylococcus, Micrococcus, Kocuria, Corynebacterium*), and scattered gut-affiliated genera (*Bacteroides, Barnesiella, Butyricimonas*) (**Figure 8**). This heterogenous profile is typical of the immediate postnatal gastric niche and does not resemble the stable oral microbiota of the mother.

No significant differential-abundance signals were detected for the newborn-placenta vs. mother-placenta contrast (p adj ≥ 0.1), consistent with the low-biomass nature of both tissues and the absence of a stable, reproducible taxonomic signature in placental samples.

## 4 Discussion

The primary finding of this study is the absence of significant differences in microbiota composition between late preterm and term pregnancies across all analyzed niches. This suggests that microbial community structure in these environments is not strongly influenced by gestational age within the studied range. The investigation of neonatal microbiota is particularly challenging, as low-biomass samples are highly susceptible to background contamination, which may be erroneously interpreted as components of the infant microbiome (21). This perspective is reflected in numerous studies. However, they consistently highlight the increased risk of contamination associated with vaginal delivery (22–26).An additional limitation is the substantially restricted study population, resulting in part from the inclusion criterion requiring delivery by cesarean section. Nevertheless, this restriction was deliberately applied to minimize potential bias in neonatal samples associated with exposure to the maternal birth canal during vaginal delivery. This is consistent with the observations of Stupak et al., who proposed that placental tissue sampling during elective cesarean section, performed as a sterile surgical procedure with intact fetal membranes, represents the safest approach for obtaining placental samples (27). Similarly, Guangyu et al. emphasized that cesarean delivery minimizes the risk of placental contamination. Nevertheless, they also noted the persistent detection of Bacteroides species, suggesting that microorganisms originating from the maternal gut, oral cavity, and vagina may potentially migrate to placental tissue regardless of the mode of delivery (28).

Accordingly, in the present study, we aimed to identify and analyze key findings encompassing both maternal and neonatal microbiota. This approach is consistent with observations reported by Bajorek et al., who suggested that neonatal exposure to the maternal birth canal during vaginal delivery may influence the colonization of gastric fluids (29).

1. Neonatal microbiota according to gestational age.

In the presented study, we obtained data on the microbiota most frequently identified at different anatomical sites in neonates. Our results indicate that LP neonates (34–36 + 6 weeks) exhibit microbiota profiles that closely resemble those of term neonates. This is consistent with previous reports suggesting that meaningful microbiota-related differences are primarily observed in infants born before 33 weeks of gestation.

Kang et al. reported that two phyla, Bacteroidota and Bacillota, constituted the majority of the neonatal meconium microbiota, and with increasing gestational age, a significant increase in the relative abundance of Bacteroidota and a decrease in Pseudomonadota were observed (30). At the genus level, *Prevotella* and *Bacteroides* were identified as dominant taxa, with *Prevotella* accounting for approximately 20–30% of the gut microbiome. In contrast, in healthy adults, the two major phyla— Bacillota and Bacteroidota —constitute over 90% of the intestinal microbiota, followed by Actinobacteriota and Pseudomonadota (31, 32).

Kloppa et al. demonstrated that gestational age is significantly associated with the composition of meconium in extremely preterm infants, with the most abundant phyla including Bacillota, Bacteroidota, Pseudomonadota, and Actinobacteriota (33). Similar findings have been reported in studies analyzing samples from neonates, which revealed differences between the gut microbiota of term and preterm infants (34–42).

Sood et al. reported that during the first week of life, the gut microbiome of term neonates is characterized predominantly by microbial signals assigned to Actinobacteriota (including *Bifidobacterium*), Pseudomonadota, and Bacteroidota, with Bacillota (including *Lactobacillus* spp., which dominate the vaginal microbiota) present at lower relative abundance (38). In contrast, Arboley et al. observed that preterm infants, compared to term neonates, exhibit increased abundance of *Enterococcus, Enterobacter*, *Lactobacillus*, and *Staphylococcus*, alongside reduced levels of *Bacteroides, Bifidobacterium*, and *Atopobium* (39). They also demonstrated that the detection of *Bifidobacterium* -related microbial signals is delayed in preterm infants (43).

A prospective study further indicated that gestational age significantly influences the presence of bifidobacterial microbial signals, with the delivery before 33 weeks of gestation being associated with reduced *Bifidobacterium*-related signals and an increased risk of infections and gastrointestinal diseases in preterm infants. Furthermore, Bartnicka et al. reported that *Bifidobacterium, Lactobacillus*, and *Streptococcus* predominate in term neonates, whereas *Enterobacteriaceae* and *Clostridium* are more prevalent in preterm infants (34).

The above observations appear to corroborate the results obtained in our study. Previous authors have highlighted differences between the microbiota of term neonates and those born before 33 weeks of gestation. In thepresented study, which included infants born after 34 weeks of gestation, as well as term neonates, no significant differences were observed between the LP and TP groups. We also identified the presence of *Bacteroides, Barnesiella, Butyricimonas*, and *Gemella* in the neonatal rectum. This may suggest the absence of major differences in the microbiota between late preterm and term neonates.

Nevertheless, these findings do not contradict reports in the literature indicating distinct microbiota in neonates born at extreme prematurity. Furthermore, we observed similarities in the presence of microbial signals in of Incertae Sedis groups between the fetal side of the placenta and the rectum, as well as of *Staphylococcus* between the placenta and the stomach.

2. Maternal and neonatal microbiota.

Due to the methodologies previously employed, which did not distinguish between live and dead bacteria and did not allow for maternal blood sampling to determine whether the microbiome originated from the maternal or fetal side of the placenta, it was generally assumed until 2014 that the placenta lacked its own microbiome (9). Current studies, however, suggest that bacterial infections are a primary cause of preterm premature rupture of membranes (PPROM) and preterm birth (10–12). It has been demonstrated that the placenta harbors its own healthy microbiome, particularly including Bacillota, Mycoplasmatota, Pseudomonadota, Bacteroidota, and Fusobacteriota. Moreover, the placental microbiome has been reported to be most similar to the human oral microbiome (37). However, the presented data did not replicate this observation. In our cohort, placental samples did not show meaningful similarity to the maternal oral (cheek) microbiota, which aligns with their clear separation in beta-diversity analyses. This discrepancy may reflect the inherently low-biomass nature of placental tissue, where microbial signals approach the detection threshold and are particularly vulnerable to background contaminants. Consequently, the lack of a placenta-oral signature in our study should be interpreted with caution and does not contradict the broader debate regarding the existence and origin of the placental microbiome.

In our study, the maternal cervix and cheek displayed distinct microbial community structures, consistent with their clearly separated clustering in beta-diversity analyses. Although both niches contained low-abundance environmental taxa, their overall compositions did not indicate direct similarity. In the neonatal population, the placental microbiota was concordant with the rectum (Incertae Sedis groups) and the stomach (*Staphylococcus*). Stout et al. similarly reported the presence of intracellular Gram-positive and Gram-negative bacteria within the basal plate, encompassing the tissue layer at the maternal–fetal interface and beneath it (44).

The literature describes theories regarding the origin of the uterine microbiota, suggesting both ascending microbial transmission from the vagina and translocation from the gut via filtration from intestinal vessels to the peritoneal cavity with reabsorption through the fallopian tubes, as well as via dendritic cells and leukocytes transporting hematogenous material to the uterus (5). Numerous reports indicate that amniotic fluid from preterm births can be colonized by microbes ascending from the vagina, even with intact membranes, and by the placental microbiome (6). Simultaneously, the vagina may serve as a source of microbes that reach the placenta, amniotic fluid, and fetus through translocation across the chorion (7, 8).

Consistent with these findings, we also observed a correlation between the cervical microbiota and the fetal side of the placenta regarding *Lactobacillus* microbial signal. However, we did not detect this pathogen in either the amniotic fluid or the neonate. The above aligns with the findings of Dobbler et al., who reported that the maternal vaginal microbiome shows limited similarity to the initial microbial signals detected in the infant gut, and that clusters of *Lactobacillus* in the maternal vagina are not associated with the presence of *Lactobacillus* in neonatal meconium at birth (45). Similarly, Park et al. suggested that the presence of *Lactobacillus*-related microbial signals in neonates is primarily associated with the natural mode of delivery.

Furthermore, the microbiota identified in the neonatal stomach in our study is noteworthy, considering that cesarean delivery was an inclusion criterion. The presence of *Staphylococcus* and *Corynebacterium* in neonatal gastric samples is largely consistent with the observations of Park et al., who reported that the microbiota of neonates delivered via cesarean section is dominated by *Staphylococcus*, *Corynebacterium*, and *Propionibacterium*, commonly found on maternal skin surfaces (46). Importantly, the neonatal stomach exhibited an exposure-driven, heterogeneous taxonomic profile rather than a stable microbial signal pattern, which is consistent with the early postnatal environment and the inherently transient nature of microbial signals detected in this niche.

According to Bartnicka et al., the application of advanced molecular techniques allows the detection of bacteria in amniotic fluid, umbilical cord blood, placenta, and fetal membranes. Moreover, the presence of specific bacterial species in meconium, such as *Escherichia coli*, *Enterococcus faecium*, and *Staphylococcus epidermidis*, may result from maternal hematogenous translocation. This hypothesis is supported by the isolation of *Enterococcus*, *Streptococcus*, *Staphylococcus*, and *Propionibacterium* species from umbilical cord blood (34).

In this context, our findings appear consistent with the notion of hematogenous translocation. The association of maternal fecal microbiota with the neonatal rectum (*Bacteroides, Barnesiella, Butyricimonas*) and maternal cheek microbiota with the neonatal rectum (*Gemella*) observed in our study is consistent with a potential association but does not demonstrate a causal mechanism.

### 4.1 Limitations

The presented study has several limitations that should be considered when interpreting the results. First, the sample size was modest, which reduces statistical power and may have limited the ability to detect subtler differences in community composition. Given the limited sample size, the study may be underpowered to detect subtle differences between cohorts, and the possibility of type II error cannot be excluded. Therefore, the absence of significant differences between LP and TP groups should be interpreted with caution. The present study should be considered exploratory and hypothesis-generating. Second, several of the analyzed niches, especially placental and neonatal stomach samples, represent low-biomass environments, where microbial signals approach the detection threshold and are inherently more susceptible to background noise and technical variability. Therefore, microbial signals detected in low-biomass samples should be interpreted with caution, as they may reflect a combination of true biological signal and background contamination. Third, although all samples were profiled using standardized 16S rRNA gene sequencing, the amplicon-based approach inherently restricts taxonomic resolution and does not allow functional inference, limiting our ability to characterize strain-level or metabolic differences. Importantly, 16S rRNA gene sequencing detects bacterial DNA and does not distinguish between viable microorganisms, transient bacterial components, or background contamination. Fourth, the inclusion of two clinically distinct newborn cohorts introduces biological heterogeneity that may not be fully captured through compositional analyses alone. Finally, as with all cross-sectional designs, causal relationships cannot be inferred, and longitudinal sampling would be required to assess temporal stability and developmental trajectories of maternal and neonatal microbiota. The study design does not allow direct inference of maternal-fetal microbial transmission pathways.

### 4.2 Future perspectives

The above data, together with reports from the literature, may provide a foundation for further research and academic considerations on the following topics: (1) the influence of the vaginal microbiota on the development of inflammatory processes in the placenta, potentially leading to preterm birth, without affecting the microbiota of the fetus or neonate born from an uncomplicated pregnancy without PPROM/PROM; (2) the impact of maternal gastrointestinal microbiota on the establishment of eubiosis in the neonatal gut, with implications for the prevention of infections and proper immunological programming, thereby reducing the risk of inflammatory conditions such as early-onset sepsis (EOS), late-onset sepsis, and necrotizing enterocolitis, as well as later-life disorders including chronic and autoimmune diseases (e.g., asthma, allergies, obesity, and diabetes); (3) the association between vaginal microbial signals related to pathogens implicated in EOS and gestational age, with particular attention to *Escherichia coli* in EOS among preterm infants and *Streptococcus agalactiae* in term neonates.

## 5 Conclusion

Most studies in the literature focus on the microbiota of extremely preterm or very immature neonates compared to term infants. In contrast, our study aimed to explore this topic by comparing late preterm neonates with term neonates.

Our analysis demonstrated no significant differences in microbiota between late preterm and term pregnancies in both mothers and neonates.

These findings suggest a possible association with microbial signals consistent with an ascending vaginal contribution; however, they do not provide direct evidence of microbial colonization or transmission. Similarly, observed patterns may point toward a potential association with maternal microbiota consistent with a hematogenous contribution, but no causal mechanism can be inferred. Therefore, these observations should be considered hypothesis-generating.

## Data Availability

The datasets presented in this study can be found in online repositories. The data that support the findings of this study were deposited at BioProject repository under accession number PRJNA 1450068.

## Supplementary material

Supplementary Table 1 Differentially abundant genera in low-biomass niches (LP vs TP).
