# Supplementary material for: Niche-driven microbial architecture in mothers and newborns with minimal cohort influence across anatomically distinct sites
Source: Front Immunol. 2026 Jul 17;17:1896420. doi: 10.3389/fimmu.2026.1896420 (PMC13423660; doi:10.3389/fimmu.2026.1896420)
Supplement: Supplementary file 2 [file DataSheet1.docx]

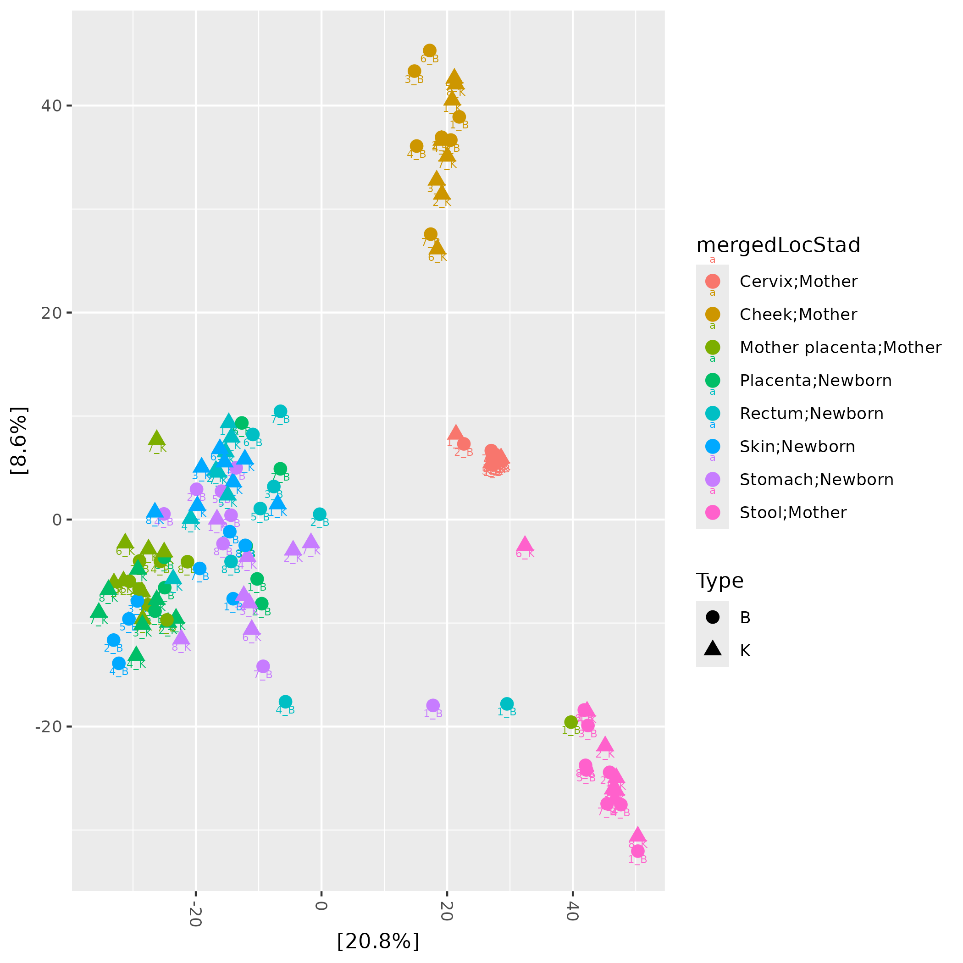


Supplementary Figure S1 (PCA).Principal Component Analysis (CLR-transformed data, Aitchison distance) showing global relationships among all maternal and neonatal niches. Samples cluster primarily by anatomical location rather than by sequencing series (LP vs TP), confirming the absence of batch effects. The distribution pattern recapitulates PCoA-based ordination, with clear separation of mother-cheek and mother-stool a compact low-biomass cluster of mother-placenta, and partially overlapping but distinct neonatal niches (rectum, skin, stomach, placenta).
